# Prevalence of *Mycobacterium tuberculosis* in Sputum and Reported Symptoms Among Clinic Attendees Compared With a Community Survey in Rural South Africa^[Author-notes ciab970-FM1]^

**DOI:** 10.1093/cid/ciab970

**Published:** 2021-11-22

**Authors:** Indira Govender, Aaron S Karat, Stephen Olivier, Kathy Baisley, Peter Beckwith, Njabulo Dayi, Jaco Dreyer, Dickman Gareta, Resign Gunda, Karina Kielmann, Olivier Koole, Ngcebo Mhlongo, Tshwaraganang Modise, Sashen Moodley, Xolile Mpofana, Thumbi Ndung’u, Deenan Pillay, Mark J Siedner, Theresa Smit, Ashmika Surujdeen, Emily B Wong, Alison D Grant

**Affiliations:** TB Centre, London School of Hygiene and Tropical Medicine, London, United Kingdom; Clinical Research Department, Africa Health Research Institute, Somkhele, South Africa; TB Centre, London School of Hygiene and Tropical Medicine, London, United Kingdom; Clinical Research Department, Africa Health Research Institute, Somkhele, South Africa; Department of Medical Statistics, London School of Hygiene and Tropical Medicine, London, United Kingdom; TB Centre, London School of Hygiene and Tropical Medicine, London, United Kingdom; Department of Medicine, University of Cape Town, Cape Town, South Africa; Clinical Research Department, Africa Health Research Institute, Somkhele, South Africa; Clinical Research Department, Africa Health Research Institute, Somkhele, South Africa; Clinical Research Department, Africa Health Research Institute, Somkhele, South Africa; Clinical Research Department, Africa Health Research Institute, Somkhele, South Africa; School of Nursing and Public Health, University of KwaZulu-Natal, Durban, South Africa; Institute for Global Health and Development, Queen Margaret University, Edinburgh, United Kingdom; TB Centre, London School of Hygiene and Tropical Medicine, London, United Kingdom; Clinical Research Department, Africa Health Research Institute, Somkhele, South Africa; Clinical Research Department, Africa Health Research Institute, Somkhele, South Africa; Clinical Research Department, Africa Health Research Institute, Somkhele, South Africa; Clinical Research Department, Africa Health Research Institute, Somkhele, South Africa; Clinical Research Department, Africa Health Research Institute, Somkhele, South Africa; Clinical Research Department, Africa Health Research Institute, Somkhele, South Africa; School of Public Health, Harvard Medical School, Boston, Massachusetts, USA; Clinical Research Department, Africa Health Research Institute, Somkhele, South Africa; Division of Infection and Immunity, University College London, London, United Kingdom; Clinical Research Department, Africa Health Research Institute, Somkhele, South Africa; Division of Infectious Diseases, Massachusetts General Hospital, Boston, Massachusetts, USA; Clinical Research Department, Africa Health Research Institute, Somkhele, South Africa; Clinical Research Department, Africa Health Research Institute, Somkhele, South Africa; Clinical Research Department, Africa Health Research Institute, Somkhele, South Africa; Division of Infection and Immunity, University College London, London, United Kingdom; Division of Infectious Diseases, Massachusetts General Hospital, Boston, Massachusetts, USA; Division of Infectious Diseases, University of Alabama Birmingham, Birmingham, Alabama, USA; TB Centre, London School of Hygiene and Tropical Medicine, London, United Kingdom; Clinical Research Department, Africa Health Research Institute, Somkhele, South Africa; School of Laboratory Medicine and Medical Sciences, College of Health Sciences, University of KwaZulu-Natal, Durban, South Africa; School of Public Health, University of the Witwatersrand, Johannesburg, South Africa

**Keywords:** culture-positive, prevalence, South Africa, sputum, tuberculosis

## Abstract

**Background:**

Tuberculosis (TB) case finding efforts typically target symptomatic people attending health facilities. We compared the prevalence of *Mycobacterium tuberculosis* (*Mtb*) sputum culture-positivity among adult clinic attendees in rural South Africa with a concurrent, community-based estimate from the surrounding demographic surveillance area (DSA).

**Methods:**

Clinic: Randomly selected adults (≥18 years) attending 2 primary healthcare clinics were interviewed and requested to give sputum for mycobacterial culture. Human immunodeficiency virus (HIV) and antiretroviral therapy (ART) status were based on self-report and record review. Community: All adult (≥15 years) DSA residents were invited to a mobile clinic for health screening, including serological HIV testing; those with ≥1 TB symptom (cough, weight loss, night sweats, fever) or abnormal chest radiograph were asked for sputum.

**Results:**

Clinic: 2055 patients were enrolled (76.9% female; median age, 36 years); 1479 (72.0%) were classified HIV-positive (98.9% on ART) and 131 (6.4%) reported ≥1 TB symptom. Of 20/2055 (1.0% [95% CI, .6–1.5]) with *Mtb* culture-positive sputum, 14 (70%) reported no symptoms. Community: 10 320 residents were enrolled (68.3% female; median age, 38 years); 3105 (30.3%) tested HIV-positive (87.4% on ART) and 1091 (10.6%) reported ≥1 TB symptom. Of 58/10 320 (0.6% [95% CI, .4–.7]) with *Mtb* culture-positive sputum, 45 (77.6%) reported no symptoms. In both surveys, sputum culture positivity was associated with male sex and reporting >1 TB symptom.

**Conclusions:**

In both clinic and community settings, most participants with *Mtb* culture-positive sputum were asymptomatic. TB screening based only on symptoms will miss many people with active disease in both settings.

## Teaser

In primary healthcare facilities and the surrounding community in rural South Africa, most people with *Mycobacterium tuberculosis*in sputum reported no symptoms. Tuberculosis case finding restricted to symptom screening in health facilities will miss many people with active disease.

South Africa is a high tuberculosis (TB)-burden country with a human immunodeficiency virus (HIV)–driven epidemic [1]. The most recent estimated incidence rate is approximately 615 per 100 000 per year; 58% of all people with TB are living with HIV [2]. While increased access to antiretroviral therapy (ART) has contributed to improved TB prevention and care, TB remains the leading cause of death in South Africa [3, 4]. Tuberculosis case finding policy in most high-TB prevalence settings recommends routine symptom screening of all adult clinic attendees and testing those who self-present with TB symptoms (cough of any duration, night sweats, loss of weight, or fever) [5, 6]. However, difficulties with facility-based case finding result in delays and missed opportunities for case detection [7–10].

Despite broad access to Xpert MTB/RIF and more recently Xpert MTB/RIF Ultra (Cepheid), the national TB prevalence in South Africa was estimated to be 737 per 100 000 in 2018 [11]. Given the impact of the coronavirus disease 2019 (COVID-19) pandemic on TB detection, there is a clear need for earlier identification and treatment of people with active TB [2, 12]. We conducted a survey at 2 primary healthcare clinics (PHCs) in KwaZulu-Natal, South Africa, within the Africa Health Research Institute (AHRI) demographic surveillance area (DSA) [13], to estimate the prevalence of *Mycobacterium tuberculosis* (*Mtb*) culture-positive sputum among adult clinic attendees. During the same period, a population-wide, community-based survey was being conducted in the DSA that included screening and testing adults for TB [14]. In this analysis we compared the prevalence of sputum culture-positivity between individuals attending clinics for ambulatory care and individuals in the surrounding community.

## METHODS

### Ethics Statement

The ethics committees of the University of KwaZulu-Natal and the London School of Hygiene and Tropical Medicine granted approval for both surveys. The Partners Human Research Committee Institutional Review Board of Partners HealthCare in the United States approved the community-based survey. Data linkage was conducted under ethics approval for the AHRI DSA [13].

All enrolled participants gave written consent or witnessed verbal consent for those who could not read or write.

### Study Design

#### Clinic Survey

In a cross-sectional study between June 2018 and May 2019, adults (aged ≥18 years) attending for healthcare were randomly selected from 2 PHCs in the Hlabisa subdistrict of uMkhanyakude, using AHRI’s electronic patient registration system as a sampling frame [15]. Exclusion criteria included attending for an emergency visit, being in labor, previous participation in the survey, or attending on behalf of a patient. Consenting adults completed a questionnaire focused on their reason for clinic attendance, TB history, and presence or absence of TB symptoms (as defined above), or any other symptoms (as an open question). Midupper arm circumference (MUAC) was measured, with a cutoff of less than 24 cm as a proxy indicator of low body mass index (BMI) [16]. Participants confidentially recorded their HIV and ART status on the digital tablet provided. All participants were asked to produce a single sputum specimen, which was sent to the AHRI Research Diagnostic Laboratory for culture in liquid media using the Mycobacteria Growth Indicator Tube system (MGIT; Becton Dickinson Microbiology Systems) and phenotypic drug sensitivity testing (DST) using the solid agar 1% proportion method for *Mtb* complex. Any participant who reported having 1 or more TB symptom was asked to provide a second sputum specimen for testing in the public health system using Xpert MTB/RIF Ultra (Xpert Ultra; Cepheid). Participants’ clinic files were reviewed for evidence of HIV and TB testing and treatment within the 12-week period after enrollment. Additional details on the survey design, participant selection, and laboratory procedures are provided in Supplementary Methods.

#### Community Survey

During the baseline data collection period, 36 097 AHRI DSA resident adults (aged ≥15 years) were eligible for enrollment. Individuals were invited to participate in a mobile screening and multi-disease testing camp that moved through the study area [14]. We report on participants who enrolled in the first 12 months of the community survey, contemporaneous with the clinic survey. As part of a comprehensive health and treatment history, all enrolled participants were screened for TB symptoms, had a MUAC measurement taken, and unless pregnant, a digital chest radiograph, which was analyzed using version 5 of the computer-aided detection for TB (CAD4TB; Thirona, Netherlands) software [17]. Participants who reported 1 or more TB symptom, had a CAD4TB score of greater than 60 (between May and September 2018) or greater than 25 (from October 2018 onwards), or were pregnant were asked to produce sputum. The sputum specimen was divided into 2 portions in the AHRI laboratory: 1 portion was tested using Xpert Ultra and the other was cultured on MGIT. Positive cultures underwent first-line DST. All participants had blood drawn for HIV testing (Genscreen Ultra HIV Ag-Ab enzyme immunoassay; Bio-Rad). Participants with a positive HIV immunoassay had a reflex HIV-1 RNA viral load performed (Abbott RealTime HIV-1 Viral Load; Abbott, USA).

In both studies, participants’ results were reviewed by a study clinician and participants were contacted and referred to care and treatment as appropriate.

#### Study Outcomes

The primary outcome for this analysis was a sputum positive for *Mtb* on liquid culture. Participants who were unable to produce a sputum specimen were considered sputum culture negative. Only *Mtb* culture-positive cases from the community survey were compared with those in the clinic survey. A secondary analysis including Xpert Ultra results is described further in the Supplementary Methods and HIV/ART status in Supplementary Table 1.

#### Data Management and Statistical Considerations

Data were captured electronically using Research Electronic Data Capture (REDCap) tools (Vanderbilt University) hosted at AHRI [18, 19]. Data entry for both studies was done using encrypted Android tablets. Data were analyzed using Stata/IC 15.1 (StataCorp, USA) and R 3.5.0 [20].

The sample size for the clinic survey was based on a precision estimate, assuming the prevalence of TB in the general population was 1.5% [21], and among PHC attendees to be around 3%. To allow for estimation of an overall TB prevalence of 3% with a precision of ±0.8%, the study aimed to enroll 3400 adult attendees (1700 per clinic) to obtain the target sample size of 2000 participants with a sputum sample, assuming that 60% could produce a specimen. No formal sample size calculations were done for the community survey, since all resident adults were eligible to participate. However, the first 10 000 participants permitted estimation of a prevalence of *Mtb*-positive sputum of 1% with a precision of ±0.2%.

The prevalence of culture-confirmed *Mtb* and its 95% confidence interval (CI) were calculated for each survey. The Supplementary Methods describe the sensitivity analysis, data linkage for the clinic survey, and risk factor analysis for both surveys.

## RESULTS

### Clinic Survey

**Figure 1.**
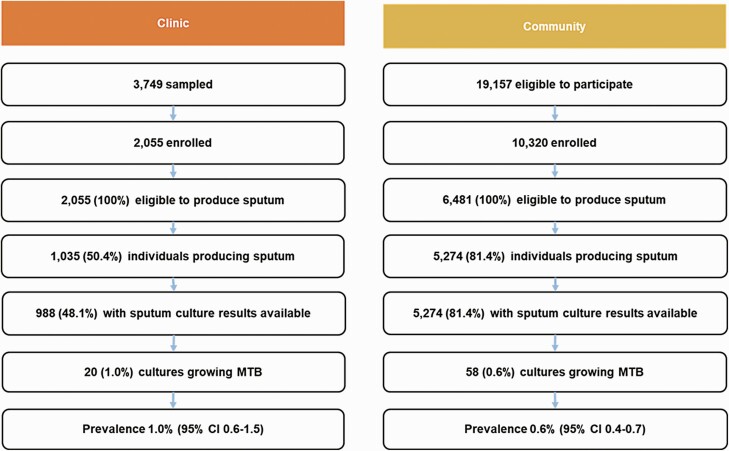
Summary of enrollment cascade for clinic and community surveys. Abbreviations: CI, confidence interval; MTB, *Mycobacterium tuberculosis*.

Between 25 June 2018 and 21 May 2019, 3506 of 7333 patients were electronically sampled and 243 were manually sampled, giving a total 3749 patients. Following screening and consent procedures, 2055 participants were enrolled into the study (Figure 1), which was fewer than originally intended because the study started later than planned. Supplementary Figure 1 illustrates the full enrollment cascade. Supplementary Table 2 compares characteristics of participants and nonparticipants among those sampled and eligible to participate, based on data linkage methods.

Among the 2055 participants enrolled, the median age was 36 (interquartile range [IQR], 28–48) years and 76.9% were female (Table 1). A total of 1479 (72%) participants were classified as HIV-positive, of whom 1463 (98.9%) were taking ART. A total of 1189 (80%) of those classified as HIV-positive reported visiting the clinic for HIV care on the day of enrollment. A total of 505 of 2055 (24.6%) participants reported past TB treatment, 14 of 2055 (0.7%) reported current TB treatment, and 131 of 2055 (6.4%) reported having 1 or more TB symptom at enrollment.

**Table 1.** Characteristics of Enrolled Participants in Clinic (n = 2055) and Community (n = 10 320) Surveys

	Clinic (n = 2055)	Community (n = 10 320)
Age, median (IQR), y	36 (28–48)	38 (23–58)
Female, n (%)	1580 (76.9)	7049 (68.3)
MUAC, median (IQR), cm	26.0 (25.0–26.0)	27.0 (24.0–30.0)
Previously treated for TB, n (%)	505 (24.6)	1228 (11.9)
On TB treatment at enrollment, n (%)	14 (0.7)	45 (0.4)
HIV status,^a^ n (%)		
Negative	536 (26.1)^b^	7151 (69.3)
Positive	1479 (72.0)^b^	3105 (30.1)
On ART	1463 (99.0)^b^	2714 (87.4)
≥1 TB symptom, n (%)	131 (6.4)	1091 (10.6)
Cough	83 (4.0)	717 (7.0)
Loss of weight	72 (3.5)	281 (2.7)
Night sweats	67 (3.3)	75 (0.7)
Fever	39 (1.9)	18 (0.2)
CAD4TB score >25, n (%)	…	5491 (53.2)
Pregnant, n (%)	Not recorded	328 (3.2)

Abbreviations: ART, antiretroviral therapy; CAD4TB, computer-aided detection for TB; HIV, human immunodeficiency virus; IQR, interquartile range; MUAC, midupper arm circumference; TB, tuberculosis.

Forty (1.9%) with missing/unknown HIV status in the clinic survey.

Self-report and clinical record review.

A total of 1035 of 2055 (50.4%) participants produced a sputum specimen, of which 47 were contaminated; 988 specimens were included in analysis. Twenty participants gave a sputum specimen that grew *Mtb*, giving a prevalence of 1.0% (95% CI, .6–1.5; 973 [95% CI, 630–1050] per 100 000) (Figure 1), which was not substantially altered (1.0% [95% CI, .6–1.5]; 990 [95% CI, 640–1054] per 100 000) after adjusting for nonresponse (Supplementary Table 4).

Among the 20 participants with a positive *Mtb* sputum culture, the median age was 37 years and 9 (45.0%) were male (Table 2). Thirteen of 20 participants (60.0%) reported having no history of TB, 15 of 20 (75.0%) were classified as HIV-positive (all on ART), and 6 of 20 (30.0%) reported having 1 or more TB symptom. Four (25.0%) *Mtb* isolates were found to be multidrug-resistant (resistant to rifampicin and isoniazid) and 1 (5.0%) mono-resistant to rifampicin. Three (60.0%) out of 5 sputum specimens with drug-resistant isolates were collected from participants who reported being previously treated for TB. All 20 participants with culture-positive sputum had further sputum tests at varying time points over the course of their care in the public health service, of which 12 were positive (Supplementary Table 5).

**Table 2.** Characteristics of Enrolled Participants Who Were Sputum Culture Positive and/or Sputum Xpert Ultra Positive for *Mycobacterium tuberculosis*: Clinic and Community Survey

	MGIT Positive Only		Xpert Ultra Positive Only
Characteristics	Clinic (n = 20)	Community (n = 58)	Community (n = 20)
Age, median (IQR), y	37 (32-46)	48 (30-64)	53 (43-60)
Male, n (%)	9 (45.0)	28 (48.0)	12 (60.0)
MUAC, median (IQR), cm	25.6 (23.9-26.0)	27.0 (24.0-30.0)	28.0 (26.0-30.0)
TB treatment history, n (%)			
On treatment	2 (10.0)	4 (6.9)	4 (20.0)
Previously treated	5 (25.0)	6 (10.3)	11 (55.0)
No history	13 (65.0)	48 (82.2)	5 (25.0)
HIV status, n (%)			
Negative	5 (25.0)^a^	31 (53.4)	10 (50.0)
Positive	15 (75.0)^a^	26 (44.8)	10 (50.0)
On ART	15 (100.0)^a^	21 (80.8)	9 (90.0)
≥1 TB symptom, n (%)	6 (30.0)	13 (22.4)	3 (15.0)
TB resistance profile, n (%)			
Rifampicin mono-resistance	1 (5.0)	2 (3.4)	2 (10.0)^b^
Multidrug resistance	4 (25.0)	9 (15.5)	…
Follow-up TB tests, n (%)			
Positive	12 (60.0)	…	…
Negative	8 (40.0)	…	…

Abbreviations: ART, antiretroviral therapy; HIV, human immunodeficiency virus; IQR, interquartile range; MGIT, Mycobacterial Growth Indicator Tube; MUAC, midupper arm circumference; TB, tuberculosis; Xpert Ultra, Xpert MTB/RIF Ultra assay (Cepheid).

Self-report and clinical record review.

Rifampicin resistance detected.

### Community Survey

Between 24 May 2018 and 25 May 2019, research teams visited 3195 households in the DSA and attempted to contact 19 157 individuals, 15 234 of whom were successfully contacted and invited to participate in the community survey (Figure 1). Of these, 578 declined and a further 4336 did not attend the mobile camps, leaving a total 10 320 enrolled participants. Of these, 6481 were asked to give a sputum specimen based on reporting 1 or more TB symptom (n = 1091), having a CAD4TB score above the study threshold (n = 5491), or being pregnant (n = 328) (Supplementary Table 3).

Among the 10 320 enrolled participants, the median age was 38 years, 68.3% were female, 3105 tested HIV-positive, and 2714 (87.4%) were on ART (Supplementary Table 3). A total of 1228 of 10 320 (11.9%) reported past TB treatment, 45 of 10 320 (0.4%) reported current TB treatment, and 1091 of 10 320 (10.6%) reported having 1 or more TB symptom at enrollment. A total of 5274 of 10 320 produced sputum specimens, of which 420 were contaminated, leaving 4854 included in the analysis. Fifty-eight sputum specimens cultured *Mtb* on liquid media, giving a prevalence of .6% (95% CI .4–.7%; 562 [95% CI, 420–710] per 100 000) (Figure 1), which remained the same after adjusting for nonresponse (.6% [95% CI, .4–.7%]; 562 [95% CI, 430–740] per 100 000). In addition, 20 participants had sputum specimens that were culture negative but Xpert Ultra positive (greater than trace).

Of the 58 participants with a positive *Mtb* sputum culture, the median age was 48 years and 28 (48.0%) were male (Table 2). Forty-eight of 58 participants (82.2%) reported having no history of TB, 26 of 58 (44.8%) were HIV-positive, and 21 of 26 (80.8%) were taking ART. Nine (15.5%) *Mtb* isolates were found to be multidrug-resistant and 2 (3.4%) were mono-resistant to rifampicin. Two (18%) out of 11 sputum specimens with drug-resistant isolates were from participants who reported previous TB treatment. The median age in the Xpert Ultra positive–only group was 53 years and 12 (60.0%) were male. Five of 20 participants (20.0%) reported having no history of TB, 10 of 20 (50.0%) were HIV-positive, and 9 of 10 (90%) were on ART. Rifampicin resistance was detected in 2 (10.0%) of these specimens; 1 of the 2 specimens was from a participant who reported previous treatment for TB.

The effects of including Xpert Ultra–positive and trace-positive results on study outcomes are described in Supplementary Results and Supplementary Table 4.

### Associations With *Mtb* Culture-Positive Sputum

In a univariable analysis of the clinic survey data (Table 3), *Mtb* sputum culture positivity was associated with TB symptoms (odds ratio [OR], 8.6 [95% CI, 3.0–24.6], *P* < .001 for >1 TB symptom vs none; OR, 3.0 [95% CI, .4–23.0], *P* = .299, for 1 TB symptom vs none) and male sex (OR, 2.8 [95% CI, 1.1–6.7]; *P* = .024).

**Table 3.** Univariable Analysis of Clinic-Based Survey (N = 2055), Showing Factors Associated With Being Sputum Culture Positive (n = 20)

Characteristics	No. Sputum Culture Positive/No. Total Participants (%)	OR (95% CI)	*P*
Sex			
Female	11/1580 (0.7)		
Male	9/472 (1.9)	2.8 (1.1–6.7)	.024
Not recorded	0/3		
Age			
<25 y	2/312 (0.6)		
25-44 y	12/1077 (1.1)	1.7 (.4–7.8)	.467
>44 y	6/666 (0.9)	1.4 (.3–7.0)	.676
HIV status^a^			
Negative	4/537 (0.7)		
Positive	15/1473 (1.0)	1.4 (.5–4.1)	.577
Unknown	1/45 (2.2)	3.0 (.3–27.7)	.326
MUAC			
≥24 cm	15/1822 (0.8)		
<24 cm	5/227 (2.2)	2.7 (1.0–7.5)	.056
Not measured	0/6		
TB symptoms			
None reported	13/1924 (0.7)		
Reported 1 symptom	1/47 (2.1)	3.0 (.4–23.0)	.299
Reported ≥2 symptoms	5/131 (3.8)	8.6 (3.0–24.6)	<.001

Abbreviations: CI, confidence interval; HIV, human immunodeficiency virus; MUAC, midupper arm circumference; OR, odds ratio; TB, tuberculosis.

Self-reported HIV status.

In the community survey (Table 4) male sex (OR, 2.0 [95% CI, 1.2–3.4]; *P* = .008), age older than 44 years (vs 15–24 years; OR, 2.5 [95% CI, 1.2–5.9]; *P* = .02), being HIV-positive on ART (vs HIV-negative; OR, 1.9 [95% CI, 1.1–3.2]; *P* = .024), having a MUAC of less than 24 cm (vs ≥24 cm; OR, 3.2 [95% CI, 1.7–5.7]; *P* < .001), and reporting 1 or more TB symptom (vs none; OR, 4.5 [95% CI, 1.9–9.0]; *P* < .001) were associated with sputum culture positivity; results were little changed after weighting for nonresponse. In a multivariable analysis (Table 4), male sex (OR, 2.4 [95% CI, 1.4–4.0]; *P* = .001), age older than 44 years (vs 15–24 years; OR, 2.7 [95% CI, 1.3–6.3]; *P* = .016), and being HIV-positive on ART (vs HIV-negative; OR, 2.0 [95% CI, 1.1–3.5]; *P* = .022) remained independently associated with sputum culture positivity.

**Table 4.** Univariable and Multivariable Analysis of Community-Based Survey (N = 10 320), Showing Factors Associated With Being Sputum Culture Positive (n = 58)

		Univariable Analysis				Multivariable Analysis			
Characteristics	No. Sputum Culture Positive/No. Total Participants (%)	OR (95% CI)	*P*	Weighted^a^ OR (95% CI)	*P*	OR (95% CI)	*P*	Weighted^a^ OR (95% CI)	*P*
Sex									
Female	30/7049 (0.4)	…		…		…		…	
Male	28/3271 (0.9)	2.0 (1.2–3.4)	.008	1.8 (1.3–2.7)	.002	2.4 (1.4–4.0)	.001	2.2 (1.5–3.3)	<.001
Age									
15–24 y	8/2802 (0.3)	…		…		…		…	
25–44 y	19/3189 (0.6)	2.1 (1.0–5.1)	.08	2.8 (1.6–5.2)	<.001	1.7 (.7–4.2)	.2	2.2 (1.2–4.3)	.011
>44 y	31/4329 (0.7)	2.5 (1.2–5.9)	.02	3.1 (1.8–5.7)	<.001	2.7 (1.3–6.3)	.016	3.3 (1.8–6.1)	<.001
HIV status									
Negative	31/7151 (0.4)	…		…		…		…	
Positive on ART	22/2714 (0.8)	1.9 (1.1–3.2)	.024	1.7 (1.1–2.6)	.01	2.0 (1.1–3.5)	.022	1.8 (1.2–2.8)	.008
Positive not on ART	4/391 (1.0)	2.4 (.7–6.0)	.11	1.8 (.7–3.7)	.20	2.7 (.8–7.0)	.077	2.0 (.8–4.2)	.080
MUAC									
≥24 cm	44/9379 (0.5)	…		…		…		…	
<24 cm	14/941 (1.0)	3.2 (1.7–5.7)	<.001	3.6 (2.3–5.4)	<.001	…		…	
TB symptoms								…	
None reported	45/9229 (0.5)	…		…					
Reported 1 symptom	5/717 (0.7)	1.4 (.5–3.3)	.4	1.3 (.6–2.5)	.5	…		…	
Reported >1 symptom	8/374 (2.1)	4.5 (1.9–9.0)	<.001	6.1 (3.6–9.7)	<.001	…		…	

Abbreviations: ART, antiretroviral therapy; CI, confidence interval; HIV, human immunodeficiency virus; MUAC, midupper arm circumference; OR, odds ratio; TB, tuberculosis.

Weights calculated as the inverse probability of participation in the community survey based on age, sex, and previous HIV status.

## DISCUSSION

We present simultaneous estimates of the prevalence of *Mtb* culture-positive sputum among ambulatory clinic attendees and adults in the surrounding community. Based on culture results only, the prevalence among clinic attendees was slightly higher than in the community, and CIs overlapped. In both surveys, most participants with culture-positive sputum did not report any symptoms, although reporting TB symptoms was associated with having culture-positive sputum. This has been reported in other community-based TB prevalence surveys [22–24] but not previously reported among adult clinic attendees. The degree of infectiousness of asymptomatic individuals with subclinical disease compared with symptomatic people is not known [25, 26]. Symptom screening as the entry point to case detection in the current model of care needs to be revisited, particularly if asymptomatic individuals are infectious.

In both surveys, the direction of the associations with risk factors was the same. HIV infection is a known risk factor for developing TB disease and, given the study setting, this finding is not unexpected [27]. HIV-positive persons taking ART attend health facilities relatively frequently, giving more opportunities for TB screening, but screening limited to those attending for HIV care will miss HIV-positive patients who attend clinics for other reasons [28]. In addition, symptom-based screening for TB has lower sensitivity among people who are HIV positive and on ART [29].

Tuberculosis prevalence estimates from previous studies based on exit interviews of only symptomatic adults attending PHCs in South Africa range between 3.6% [8] and 5% [9]. Our estimate of lower TB prevalence among clinic attendees in comparison to previous estimates is most likely a result of our attempt to construct a true random sample of adult ambulatory care attendees and to request sputum from all those enrolled, regardless of symptoms.

Our community survey screening methods are comparable to other surveys [22, 23, 30, 31], but our estimate is based on a single sputum specimen. National TB prevalence survey estimates are based on 2 specimens and our estimate is therefore likely to underestimate prevalence compared with the national survey and other surveys in which multiple samples are collected. Our community estimates are consistent with those reported in a recent study from Uganda based on a single sputum specimen tested with Xpert Ultra [24]. Including all Xpert Ultra–positive tests resulted in an estimated community prevalence of 940 (95% CI, 780–1130) per 100 000 and 420 (95% CI, 320–550) per 100 000 adults when Xpert Ultra trace-positive and culture-negative sputum results were excluded.

Our community survey aimed to enroll as many DSA residents as possible; our sample was therefore not representative, but weighting for nonresponse did not materially affect our estimate. In both surveys, men were underrepresented, which may have resulted in an underestimate of overall prevalence, since men are known to be disproportionately affected by TB [32, 33]. In the same way, our prevalence findings could be an overestimate if people who were ill were more likely to participate in the community survey, but this would not explain the high number of participants with culture-positive sputum who were asymptomatic at enrollment.

Chest radiography is the most sensitive TB screening method currently available [22, 23, 31, 34] and has the potential to substantially increase the yield of case finding in high-prevalence settings [35]. Although costly, digital chest radiography in combination with computer-aided detection software is a promising alternative in settings where limited human resources are a barrier to implementation [36].

Our analysis has the following limitations. Participation in both surveys was incomplete, but prevalence estimates were not substantially different after weighting for nonresponse. The clinic survey did not achieve its intended sample size, resulting in a less precise estimate than planned. Only 50% of clinic survey participants were able to produce a sputum sample, which may have resulted in an underestimate of the true prevalence of TB among clinic attendees. Because we did not request sputum from all participants in the community survey, we may have underestimated true prevalence. Due to logistical constraints, both surveys relied on a single sputum specimen from each participant to detect active TB, and our primary comparison is based on sputum MGIT culture only. Studies among patients being investigated for TB have estimated the incremental yield of culture-positive *Mtb* from a second sputum specimen to be between 6% and 10% [37, 38]. In the clinic survey, most participants with culture-positive sputum had a second specimen confirming TB disease through routine care in the public health service. Had any of the clinic survey sputum specimens been false-positive cultures, our prevalence estimate would be an overestimate, in which case the true prevalence would be closer to the community-based estimate. In addition, because of the limited number of cases, false-positive results could have biased the results of the multivariable analysis. Reliance on a single sputum specimen in the community survey could have resulted in an overestimate from false-positive results, but equally, collecting only 1 specimen may have resulted in an underestimate of true prevalence [37].

In conclusion, TB case finding based on symptom screening and restricted to health facilities will miss many people with TB disease. If individuals without symptoms, in the subclinical phase of the disease, are infectious, the existing case finding strategy will need to be reconsidered. Work towards understanding the relative contribution of asymptomatic people to TB transmission is ongoing and will be of particular importance to determine the conditions under which symptom-agnostic screening algorithms should be considered. A clear strategy is also needed to detect HIV-negative people with TB in the community. There is an urgent need for better low-cost, high-sensitivity screening tests for TB in community and clinic settings.

## Supplementary Data

Supplementary materials are available at *Clinical Infectious Diseases* online. Consisting of data provided by the authors to benefit the reader, the posted materials are not copyedited and are the sole responsibility of the authors, so questions or comments should be addressed to the corresponding author.

ciab970_suppl_Supplementary_Material

## Notes

***Acknowledgments.*** The authors thank the study participants, community, members of the community advisory board, staff in the health facilities where these studies were conducted, and the many AHRI staff who contributed to these studies, including Nkosingiphile Buthelezi, Siphephelo Dlamini, Yutu Dlamini, Anita Edwards, Patrick Gabela, Emmerencia Gumede, Sashin Harilall, Kobus Herbst, Mandla Khoza, Nozi Khumalo, Zilethile Khumalo, Nondumiso Kumalo, Richard Lessells, Sithembiso Luthuli, Sinethemba Mabuyakhulu, Nonhlanhla Madlopha, Thabile Mkhize, Duduzile Mkhwanazi, Zinhle Mkhwanazi, Zodwa Mkhwanazi, Sihle Mthethwa, Sphiwe Mthethwa, Nozipho Mthethwa, Silindile Mthembu, Sanele Mthiyane, Vanisha Munsamy, Tevania Naidoo, Nompilo Ndlela, Thandekile Nene, Sabelo Ntuli, Nompumulelo Nyawo, Anand Ramnanan, Aruna Sevakram, Sizwe Sikhakane, Zizile Sikhosana, Marlise Venter, Precious Zulu, and all the staff of the AHRI Research Diagnostic Laboratory.

***Financial support.*** The clinic survey is part of the Umoya Omuhle program, supported by the Economic and Social Research Council (ESRC) (grant number ES/P008011/1), in partnership with the National Institute for Health Research (NIHR), Arts and Humanities Research Council (AHRC), the Medical Research Council (MRC), and the Department for Environment, Food and Rural Affairs (Defra) and the Veterinary Medicines Directorate (VMD). The community survey was supported by the Wellcome Trust via a strategic award to AHRI (grant number 201433/Z/16/A).
