# Correction to: Prevalence of *Mycobacterium tuberculosis* in Sputum and Reported Symptoms Among Clinic Attendees Compared With a Community Survey in Rural South Africa

**DOI:** 10.1093/cid/ciac244

**Published:** 2022-04-27

**Authors:** Indira Govender, Aaron S Karat, Stephen Olivier, Kathy Baisley, Peter Beckwith, Njabulo Dayi, Jaco Dreyer, Dickman Gareta, Resign Gunda, Karina Kielmann, Olivier Koole, Ngcebo Mhlongo, Tshwaraganang Modise, Sashen Moodley, Xolile Mpofana, Thumbi Ndung’u, Deenan Pillay, Mark J Siedner, Theresa Smit, Ashmika Surujdeen, Emily B Wong, Alison D Grant

**Affiliations:** TB Centre, London School of Hygiene and Tropical Medicine, London, United Kingdom; Clinical Research Department, Africa Health Research Institute, Somkhele, South Africa; TB Centre, London School of Hygiene and Tropical Medicine, London, United Kingdom; Clinical Research Department, Africa Health Research Institute, Somkhele, South Africa; Department of Medical Statistics, London School of Hygiene and Tropical Medicine, London, United Kingdom; TB Centre, London School of Hygiene and Tropical Medicine, London, United Kingdom; Department of Medicine, University of Cape Town, Cape Town, South Africa; Clinical Research Department, Africa Health Research Institute, Somkhele, South Africa; Clinical Research Department, Africa Health Research Institute, Somkhele, South Africa; Clinical Research Department, Africa Health Research Institute, Somkhele, South Africa; Clinical Research Department, Africa Health Research Institute, Somkhele, South Africa; School of Nursing and Public Health, University of KwaZulu-Natal, Durban, South Africa; Institute for Global Health and Development, Queen Margaret University, Edinburgh, United Kingdom; TB Centre, London School of Hygiene and Tropical Medicine, London, United Kingdom; Clinical Research Department, Africa Health Research Institute, Somkhele, South Africa; Clinical Research Department, Africa Health Research Institute, Somkhele, South Africa; Clinical Research Department, Africa Health Research Institute, Somkhele, South Africa; Clinical Research Department, Africa Health Research Institute, Somkhele, South Africa; Clinical Research Department, Africa Health Research Institute, Somkhele, South Africa; Clinical Research Department, Africa Health Research Institute, Somkhele, South Africa; School of Public Health, Harvard Medical School, Boston, Massachusetts, USA; Clinical Research Department, Africa Health Research Institute, Somkhele, South Africa; Division of Infection and Immunity, University College London, London, United Kingdom; Clinical Research Department, Africa Health Research Institute, Somkhele, South Africa; Division of Infectious Diseases, Massachusetts General Hospital, Boston, Massachusetts, USA; Clinical Research Department, Africa Health Research Institute, Somkhele, South Africa; Clinical Research Department, Africa Health Research Institute, Somkhele, South Africa; Clinical Research Department, Africa Health Research Institute, Somkhele, South Africa; Division of Infection and Immunity, University College London, London, United Kingdom; Division of Infectious Diseases, Massachusetts General Hospital, Boston, Massachusetts, USA; Division of Infectious Diseases, University of Alabama Birmingham, Birmingham, Alabama, USA; TB Centre, London School of Hygiene and Tropical Medicine, London, United Kingdom; Clinical Research Department, Africa Health Research Institute, Somkhele, South Africa; School of Laboratory Medicine and Medical Sciences, College of Health Sciences, University of KwaZulu-Natal, Durban, South Africa; School of Public Health, University of the Witwatersrand, Johannesburg, South Africa

An error appeared in the corrected proof publication of this article [Govender I, Karat AS, Olivier S, et al. Prevalence of Mycobacterium tuberculosis in Sputum and Reported Symptoms Among Clinic Attendees Compared With a Community Survey in Rural South Africa. Clin Infect Dis https://doi.org/10.1093/cid/ciab970]. The last line of the Financial support section currently states “The community survey was supported by the Wellcome Trust via a strategic award to AHRI (grant number 201433/Z/16/Z)”. The sentence should state “The community survey was supported by the Wellcome Trust via a strategic award to AHRI (grant number 201433/Z/16/A)”.

The author regrets this error.

