# Supplementary material for: Prevalence of Mycobacterium tuberculosis in Sputum and Reported Symptoms Among Clinic Attendees Compared With a Community Survey in Rural South Africa
Source: Clin Infect Dis. 2021 Nov 22;75(2):314–22. doi: 10.1093/cid/ciab970 (PMC9410725; doi:10.1093/cid/ciab970)
Supplement: ciab970_suppl_Supplementary_Material [file ciab970_suppl_supplementary_material.docx]

**SUPPLEMENT**

[Govender et al., Prevalence of Mycobacterium tuberculosis in sputum among clinic attendees compared to a community survey in rural South Africa]

Table of Contents

[Supplemental Methods 2](#_Toc87531823)

[Clinic Survey: Study design and participants 2](#_Toc87531824)

[Laboratory procedures 2](#_Toc87531825)

[Study Outcomes 3](#_Toc87531826)

[Table S1: Defining HIV status among participants in the clinic-based survey 4](#_Toc87531827)

[Sensitivity Analysis 4](#_Toc87531828)

[Clinic Survey: Data Linkage 4](#_Toc87531829)

[Risk Factor analysis 5](#_Toc87531830)

[Supplemental Results 6](#_Toc87531831)

[Figure S1: Clinic-based survey enrolment cascade 6](#_Toc87531832)

[Table S2 - Comparison of characteristics between eligible individuals who participated in the clinic-based survey and those who did not participate 7](#_Toc87531833)

[Table S3: HIV status among participants in the clinic-based survey 8](#_Toc87531834)

[Effect of including Xpert Ultra results on study outcomes: 8](#_Toc87531835)

[Table S4: Effect of Xpert Ultra results on prevalence estimates 9](#_Toc87531836)

[Table S5: Follow-up TB test results among participants with culture-positive sputum (N = 20) 9](#_Toc87531837)

[References: 10](#_Toc87531838)

# Supplemental Methods

## Clinic Survey: Study design and participants

All registered patients had an equal chance of being randomly selected at each visit, to allow those who initially declined to participate an opportunity to enrol at a subsequent visit. When the electronic system was not functioning, the study team manually selected a systematic sample of patients in the waiting areas. After being selected, patients were screened for eligibility by the study team.

## Laboratory procedures

In the clinic survey, sputum samples were decontaminated using the *N*-Acetyl-l-Cysteine–Sodium Hydroxide (NALC/NaOH) method of decontamination. From the reconstituted decontamination sediment 0.5ml was inoculated into a Mycobacteria Growth Indicator Tube (MGIT™) and run on an automated BACTEC MGIT 960 system (Becton Dickinson, Sparks, MD). Any MGIT that was flagged positive underwent investigation via a sub-culture on blood agar, Ziehl-Neelsen staining and MGIT TBc identification test (TBc ID)(Becton Dickinson, Sparks, MD) to confirm *Mycobacterium tuberculosis* (MTB). The 1% proportional Drug Susceptibility Test (DST) was performed on all MTB positive samples. In the community survey, if the volume of sputum collected was > 2ml, a 1ml raw sputum aliquot was frozen at -80˚C.  The remainder of the sample was then decontaminated using the NALC/NaOH method of decontamination. From the reconstituted decontamination sediment 0.5ml was inoculated into a MGIT and run on a BACTEC MGIT 960 and 1ml was used for GeneXpert MTB/RIF Ultra testing (Cepheid Inc., Sunnyvale, USA). Any MGIT that was flagged positive underwent the same investigation as clinic survey samples and a further 1 ml of the MGIT was used to perform another GeneXpert MTB/RIF Ultra. The 1% proportional DST was performed on all confirmed MTB positive samples.

## Study Outcomes

In a secondary analysis, participants from the community survey with a positive Xpert Ultra result but a negative mycobacterial culture result were included in the prevalence estimate. In the clinic survey, HIV and ART status were classified according to self-report and clinical record review findings (Table S1). In the community survey, HIV and ART status were based on immunoassay for HIV and viral load, as described in the methods.

## Table S1: Defining HIV status among participants in the clinic-based survey

| Category | Definitions |
| --- | --- |
| HIV-positive | Evidence from clinical records of an HIV-positive test result; CD4 count or HIV viral load or of being on antiretroviral therapy (ART) before and up to a period of 3 months after the date of enrolment. |
|  | OR if no evidence of HIV-positive status in clinical records, self-reported being HIV-positive OR on ART. |
| HIV-positive on ART | Evidence from clinical records of being on ART before and up to a period of 3 months after the date of enrolment. |
|  | OR if no evidence of being on ART on clinical records, self-reported being on ART. |
| HIV-negative | Evidence of an HIV-negative test result at least 12 months before the date of enrolment, AND no evidence in clinical records of a CD4 count, HIV viral load or of being on ART. |
|  | OR self-reported being HIV-negative and had evidence of being HIV-positive dated more than 3 months after the date of enrolment. |
|  | OR self-reported being HIV-negative AND clinical records did not have any evidence of an HIV test result, CD4 or HIV viral load, or ART OR no clinical records available for review. |
| HIV status unknown | Self-reported having no knowledge of their HIV status AND EITHER no clinical records available for review OR the file review did not have any evidence of an HIV test result, CD4 count, HIV viral load or ART. |
|  | OR self-reported being HIV-negative AND only evidence of an HIV-negative test is at least 12 months before enrolment. |

## Sensitivity Analysis

As a sensitivity analysis, estimates were weighted to account for non-response; weights were calculated as the inverse probability of participation, using a logistic regression model with covariates for age and sex (both surveys) and clinic, reason for attendance, and HIV status (clinic survey only).

## Clinic Survey: Data Linkage

The HIV status of those who were sampled and eligible to participate in the clinic survey was determined by linking with data from the following sources: the community study; the annual HIV serosurvey in the DSA; previous records of clinic visits in the electronic patient registration system; and the HIV care electronic patient records system (TIER.Net) used in public PHC clinics(1,2). AHRI receives TIER.Net data from 17 clinics in the district, including those in the DSA. Individuals in TIER.Net who are members of the DSA are retrospectively linked with their surveillance identification number, using deterministic, “fuzzy”, and probabilistic record linkage algorithms.

## Risk Factor analysis

Factors (sex, age categories, HIV status, MUAC, and reporting one or more TB symptoms) potentially associated with having *Mtb* culture-positive sputum were investigated using univariable analysis for both surveys. Factors associated with the outcome in univariable analysis with p-values of less than 0.05 in the community-based survey were further analysed in a multivariable logistic regression equation. Multivariable analysis was not undertaken for the clinic survey because the number of outcomes was small.

# Supplemental Results

## Figure S1: Clinic-based survey enrolment cascade

**
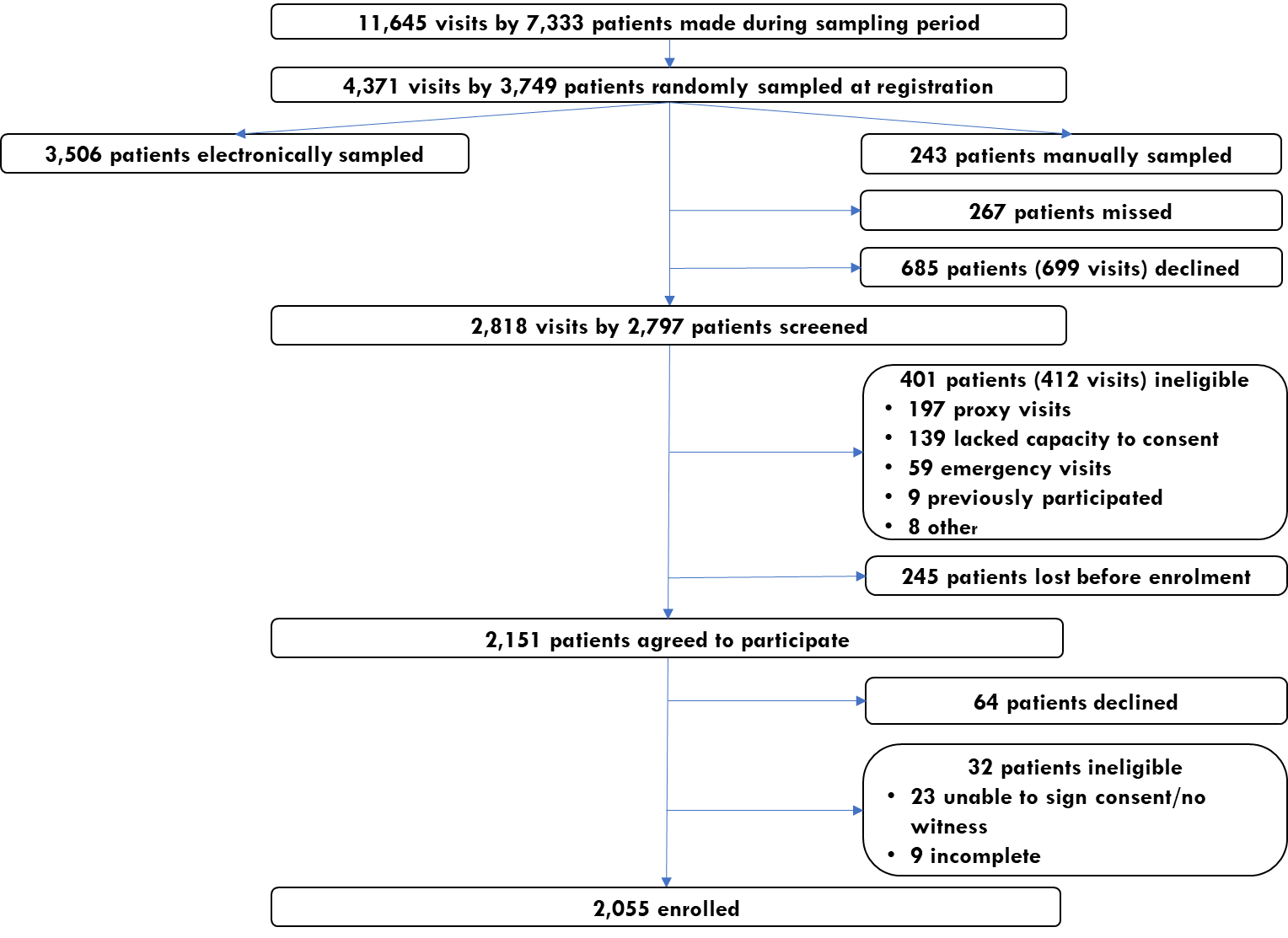
**

## Table S2 - Comparison of characteristics between eligible individuals who participated in the clinic-based survey and those who did not participate

|  | All eligible^*^, n (%) | Participated, n (%) | Did not participate, n (%) |
| --- | --- | --- | --- |
|  | 2,956 | 2,055 | 901 |
| Age group (years) |  |  | P=0.11 |
| <25 | 427 (14.4) | 312 (15.2) | 115 (12.8) |
| 25-34 | 859 (29.1) | 596 (29.0) | 263 (29.2) |
| 35-44 | 692 (23.4) | 481 (23.4) | 211 (23.4) |
| 45-54 | 459 (15.5) | 327 (15.9) | 132 (14.7) |
| ≥54 | 519 (17.6) | 339 (16.5) | 180 (20.0) |
| Sex |  |  | P=0.001 |
| Male | 728 (24.7) | 471 (23.0) | 257 (28.5) |
| Female | 2,224 (75.3) | 1,580 (77.0) | 644 (71.5) |
| HIV status^1^ |  |  | P=0.002 |
| Positive | 1,924 (65.1) | 1,294 (63.0) | 630 (69.9) |
| Negative in 2018/2019 | 247 (8.4 ) | 167 (8.1) | 80 (8.9 ) |
| Last negative 2016/17 | 37 (1.3) | 27 (1.3) | 10 (1.1) |
| Last negative 2013/2015 | 16 (0.5) | 12 (0.6) | 4 (0.4 ) |
| Last negative ≤2012 | 13 (0.4) | 9 (0.4) | 4 (0.4 ) |
| Unknown | 719 (24.3) | 546 (26.6) | 173 (19.2) |
| Reason for clinic attendance^2^ |  |  | P<0.001 |
| HIV/TB | 1,763 (59.6) | 1,197 (58.2) | 566 (62.8) |
| Other chronic care | 365 (12.3) | 222 (10.8) | 143 (15.9) |
| Other | 574 (19.4 ) | 389 (18.9) | 185 (20.5 ) |
| Unknown (not recorded) | 254 (8.6 ) | 247 (12.0) | 7 (0.8 ) |
| *The total number of eligible individuals excludes those who were missed after being sampled and those who were ineligible based on exclusion criteria but includes those who were eligible and refused to participate.  ^1^Last known HIV status, as determined through the anonymous HIV serosurvey, the community-based survey, or a record of an ART visit in TIER.net or in electronic patient registration system. Individuals who last tested negative in the serosurvey with no subsequent record of a positive test or a record of ART care are classified as HIV negative, by year last tested. Those who never tested in the serosurvey, and had no record of ART care were classified as unknown.  ^2^Participation also differed by reason for visiting the clinic based on electronic registration data, with individuals whose reason for visit was not recorded more likely to participate than those visiting for other reasons. | | | |
| HIV, Human immunodeficiency virus; TB, tuberculosis; ART, antiretroviral therapy | | | |

## Table S3: HIV status among participants in the clinic-based survey

| Category | Definitions | N / 2055 (%) |
| --- | --- | --- |
| HIV-positive | Evidence from clinical records of an HIV positive test result; CD4 count or HIV viral load or of being on antiretroviral therapy (ART) before and up to a period of 3 months after the date of enrolment. | 1,155 (56.2) |
|  | OR if no evidence of HIV-positive status in clinical records, self-reported being HIV positive OR on ART. | 324 (15.8) |
| Total HIV-positive | | **1,479 (72.0)** |
| HIV-positive on ART | Evidence from clinical records of being on ART before and up to a period of 3 months after the date of enrolment. | 1,155 (56.2) |
|  | OR if no evidence of being on ART on clinical records, self-reported being on ART. | 308 (15.0) |
| Total HIV-positive on ART | | **1,463 (71.2)** |
| HIV-negative | Evidence of an HIV negative test result at least 12 months before the date of enrolment, AND no evidence in clinical records of a CD4 count, HIV viral load or of being on ART. | 46 (2.2) |
|  | OR self-reported being HIV negative and had evidence of being HIV positive dated more than 3 months after the date of enrolment. | 2 (0.1) |
|  | OR self-reported being HIV negative AND clinical records did not have any evidence of an HIV test result, CD4 or HIV viral load, or ART OR no clinical records available for review. | 488 (23.7) |
| Total HIV-negative | | **536 (26.1)** |
| HIV status unknown | Self-reported having no knowledge of their HIV status AND EITHER no clinical records available for review OR the file review did not have any evidence of an HIV test result, CD4 count, HIV viral load or ART. | 37 (1.8) |
|  | OR self-reported being HIV negative AND only evidence of an HIV negative test is at least 12 months before enrolment. | 3 (0.1) |
| Total HIV status unknown | | **40 (1.9)** |
| HIV, Human Immunodeficiency Virus | | |

## Effect of including Xpert Ultra results on study outcomes:

When the 20 positive Xpert Ultra results were included in the estimate, the community-based survey prevalence increased to 0.8% (95% CI 0.6–1.0%; 760 [95% CI 590–920] per 100,000) and remained the same when weighted for non-response (0.8% [95% CI 0.6–1.0]; 750 [95% CI 590–950] per 100,000; Table S3). 32 participants had only trace-positive results on Xpert Ultra and were not classified as positive for *Mtb* in this analysis. Had trace-positive results been included with positive Xpert Ultra results in the analysis, the prevalence estimate for the community-based survey would have been 1.0% (95% CI 0.8–1.2; 1,020 [95% CI 820–1,210] per 100,000) and not materially altered when weighted for non-response (1.0% [95% CI 0.8–1.2]; 990 [95% CI 810–1,220] per 100,000).

## Table S4: Effect of Xpert Ultra results on prevalence estimates

|  | Clinic prevalence, n (% [95% CI]) | Community prevalence, n (% [95% CI]) |
| --- | --- | --- |
| **MGIT culture only** | 20 (1.0 [0.6-1.5]) | 58 (0.6 [0.4-0.7]) |
| **MGIT culture only weighted for non-response** | 20 (1.0 [0.6-1.5]) | 58 (0.6 [0.4-0.7]) |
| **MGIT culture + positive Xpert Ultra results, excluding trace positives** | - | 78 (0.8 [0.6-1.0]) |
| **MGIT culture + positive Xpert Ultra results, excluding trace positives, weighted for non-response** | - | 78 (0.8 [0.6-1.0]) |
| Xpert Ultra, Xpert® MTB/RIF Ultra assay; MGIT, Mycobacterial Growth Indicator Tube; CI, confidence interval | | |

## Table S5: Follow-up TB test results among participants with culture-positive sputum (N = 20)

| Test | Positive | Negative |
| --- | --- | --- |
| Xpert® MTB/RIF Ultra | 10 | 2 |
| Sputum microscopy | 1 | 3^*^ |
| Sputum culture | 1 | 3^*^ |
| ^*^Two of the negative microscopy tests and one of the negative culture tests were done between 2 to 5 months after the participant initiated TB treatment. | | |

# References:

1. Gareta D, Baisley K, Mngomezulu T, et al. Cohort Profile Update: Africa Centre Demographic Information System (ACDIS) and population-based HIV survey. Int J Epidemiol. 2020; 50: 33–34.
2. Osler M, Hilderbrand K, Hennessey C, et al. A three-tier framework for monitoring antiretroviral therapy in high HIV burden settings. J Int AIDS Soc. 2014 ; 17 :18908.
